# Fragment-Based Screening by Protein Crystallography: Successes and Pitfalls

**DOI:** 10.3390/ijms131012857

**Published:** 2012-10-08

**Authors:** Zorik Chilingaryan, Zhou Yin, Aaron J. Oakley

**Affiliations:** School of Chemistry, University of Wollongong, Northfields Ave, Wollongong 2522, NSW, Australia; E-Mails: zc902@uowmail.edu.au (Z.C.); zy877@uowmail.edu.au (Z.Y.)

**Keywords:** fragment-based screening, crystallography, drug design, synchrotron radiation, X-ray

## Abstract

Fragment-based drug discovery (FBDD) concerns the screening of low-molecular weight compounds against macromolecular targets of clinical relevance. These compounds act as starting points for the development of drugs. FBDD has evolved and grown in popularity over the past 15 years. In this paper, the rationale and technology behind the use of X-ray crystallography in fragment based screening (FBS) will be described, including fragment library design and use of synchrotron radiation and robotics for high-throughput X-ray data collection. Some recent uses of crystallography in FBS will be described in detail, including interrogation of the drug targets β-secretase, phenylethanolamine *N*-methyltransferase, phosphodiesterase 4A and Hsp90. These examples provide illustrations of projects where crystallography is straightforward or difficult, and where other screening methods can help overcome the limitations of crystallography necessitated by diffraction quality.

## 1. Introduction

Contemporary drug discovery efforts are aimed at modulating the activities of specific targets (almost always a protein that is essential to a pathogen, or a human protein that is misregulated, misfolded or mutated). The identification of chemical leads against these targets is a key step in the drug discovery process. Starting-points for chemical leads include natural products, high throughput screening (HTS) of large chemical libraries, and most recently fragment-based drug discovery (FBDD). The latter is a method that has evolved over the past ~20 years for generating high affinity ligands to serve as starting points for the development of drug candidates [1]. The FBDD approach utilizes compounds of lower molecular weight (<~300 Da) compared to those used in high-throughput screening. The origins of FBDD are debatable, but it has been documented [2] that X-ray crystallography was first used to map the interactions of small-molecule organic solvents (e.g., acetonitrile and isopropanol) on protein surfaces [3,4]. In 1996, Shuker and co-workers [5] described “SAR by NMR”, in which NMR was used to map small organic molecules to sub-sites on a protein, followed by the optimization and linking of these entities to produce high-affinity ligands. In 1998, Stout and co-workers demonstrated the additive nature of substrate fragments in crystal structures of thymidylate synthase in complex with fragments of deoxyuridine monophosphate [6], suggesting a modular approach to lead design. By the 2000s, these small organic molecules were referred to as “fragments” [7], “binding elements” [8] “needles” [9], “shapes” [10], or “seed templates” [11]. Notably, approaches using FBDD have been successful when large HTS screens have failed, for example, in the development of β-secretase (BACE) inhibitors [12].

FBDD differs with respect to the more established HTS in several aspects. The diversity of chemical functionality that can be sampled relative to the volume of chemical space is greater for fragments, giving an elevated hit-rate compared with HTS. As ligands become more complex, the probability of observing relevant interactions falls dramatically for a given library [13]. A consequence of higher chemical diversity and better hit-rates is that fragment libraries tend to be smaller in size (in the order of 10^3^ compounds) compared with libraries of larger compounds used in HTS (which may contain up to 10^6^ compounds). The smaller size of fragment libraries compared with HTS libraries makes FBDD accessible to small biotechnology companies and academic laboratories that do not have access to large compound libraries. Fragments tend to have low affinity for their targets compared with HTS hits, and fragment-screening techniques need sufficient sensitivity to detect hits with *K*_d_ values in the mM to high μM range. This low affinity is partly a consequence of overcoming a rigid body entropic barrier, estimated to be 15–20 kJ/mol (or 3 orders of magnitude in *K*_d_) at 298 K [14]. This effect is independent of molecular weight and thus fragments that overcome this barrier form “high quality” interactions (highly energetically favorable interactions that overcome the entropic cost of binding). Fragments often bind with better “ligand efficiencies” (LE) than traditional screening hits [15], where LE is a function of binding free energy and the number of heavy atoms (*N*_HA_) in the system: LE = −Δ*G*/*N*_HA_ [16]. An important aspect of the optimization process is that, as fragments are developed into leads, these “high quality” interactions are maintained [17]. Thus, in the mature lead compound it is possible to see moieties of the fragment from which the lead grew.

Early FBDD projects utilized crystallography [7] or NMR [10] methods as primary screening methods. Validation of hits is a vital component of the FBDD strategy and should include a technique to estimate binding affinity. Isothermal titration calorimetry (ITC) is considered by some [17] as the “gold standard” for validation. ITC is highly sensitive and can be used to determine the enthalpic and entropic contributions of a fragment to the binding free energy. Boehm and co-workers [9] used *in silico* screening followed by validation by biophysical methods including NMR for targeting DNA Gyrase. More recently, thermal shift assays (TSA) [18] and surface plasmon resonance (SPR) [19] have been employed.

In this review, we shall address practical considerations in FBS by crystallography and provide examples of its use in successful drug discovery programs, highlighting cases where complementary approaches have assisted the discovery process, and some potential pitfalls.

## 2. Practical Considerations in FBDD

### 2.1. Library and Compound Properties

Lead compounds must have high affinity for the target, and “drug like” physico-chemical and pharmacodynamic properties. Lipinski and co-workers [20] identified key features of orally bioavailable drugs in what is now called the “rule of five”. These are: molecular weight < 500 Da; calculated log partition coefficient between octanol and water (clogP, a measure of lipophilicity) < 5; number of hydrogen bond donors ≤ 5; number of hydrogen bond acceptors ≤ 10. Congreve and coworkers analyzed a diverse set of fragment hits against a range of targets and developed the so-called “rule of three” [21]. These are: molecular weight < 300 Da; clogP ≤ 3; number of hydrogen-bond donors ≤ 3; number of hydrogen-bond acceptors ≤ 3. While fragment libraries are designed with functional group diversity in mind, reactive and potentially toxic functional groups unsuitable for drugs are excluded [12]. Fragment libraries tend to be biased toward planar, achiral heterocycles and it has been argued recently that the use of fragments richer in *sp*^3^-centres should result in increased hits on distinct sites on biological targets [22].

Size estimates of chemical space vary greatly depending on the criteria used. An early (1996) study on the size of chemical space (*i.e.*, the number of possible molecules) with drug-like properties was estimated to be as high as 10^60^ compounds [23]. A recent, much lower estimate based on a power-function derived from the growth of organic compounds as a function of the number of carbon atoms puts the size of chemical space of drug-like compounds with ≤100 carbon atoms at 3.4 × 10^9^ [24]. Fink and Reymond estimated that the number of chemical entities with up to 11 C, N, O and F atoms that follow Congreve’s rule of three to be 13.2 million [25].

Owing to their inherently weak binding, fragments need to be soluble at about 10 mM in crystal soak buffers from stock solutions in dimethylsulfoxide (DMSO) at about 0.1 M. To improve the efficiency of fragment soaking, cocktails containing multiple compounds are used. The number of fragments in the cocktail is dictated by the required concentration and the concentration of DMSO tolerated by the crystals.

Several commercial fragment libraries suitable for crystallographic screening are available. Zenobia Therapeutics (San Diego, CA, http://www.zenobiatherapeutics.com) distributes two sets of compounds “Library 1” (352 compounds) and “Library 2” (286 compounds). Maybridge (Cornwall, UK, http://www.maybridge.com) distributes the Ro3 (Rule of 3) 2500 Diversity Fragment Library consisting of a “core set” of 1000 compounds with a “supplement set” of 1500 compounds. Otava (Kyiv, Ukraine, http://www.otavachemicals.com) supplies fragment libraries from a set of 7129 compounds. ChemBridge (San Diego, CA, http://www.chembridge.com) distributes a >7000 compound fragment library. All libraries use the aforementioned “rule of three” criteria [21] and in several cases, additional (proprietary) filters for shape and chemistry in library development. Information supplied with the libraries varies with suppliers but will generally include chemical structures with SMILES strings [26] and three-dimensional structure files.

### 2.2. The Target

The most important requirement for crystallographic FBS is the availability of target protein crystals for which a structure solution is available. Crystals must be robust, stable under soaking conditions and diffract to beyond about 2.5 Å resolution—sufficient to place fragments unambiguously in electron density. Crystallization still represents a significant bottleneck in structure determination by X-ray diffraction techniques. Today, techniques and tools are available to aid in the crystallization of difficult targets. Automation in the form of robotic plate-based screening techniques or microfluidic platforms allow many more conditions to be sampled and for economical use (sub-microliter volumes) of the sample [27,28]. Furthermore, techniques in molecular biology and protein chemistry, including modification of the target by enzymes (“see below”) and protein engineering, can aid in the crystallization of difficult targets.

Proteins produced naturally or through recombinant expression may contain flexible or disordered regions that hinder the formation of crystal contacts. Possibilities include the *N*- and *C*-terminal regions, internal loops and post-translational modifications (e.g., many eukaryotic proteins undergo *N-* and *O*-glycosylation). The removal of flexible and heterogeneous glycosidic groups to improve crystallizability is a well-established strategy [29] and numerous deglycosylating enzymes are commercially available. Successful strategies have been outlined for the use of proteases to form stable fragments of proteins for crystallization. Recently, proteolysis *in situ* (addition of trace amounts of trypsin or chymotrypsin) has been reported [30]. In such cases, flexible loops or termini regions—that potentially block crystal contact formation—accessible to the protease are removed. This strategy may fail due to incomplete proteolysis leading to sample heterogeneity.

As most protein targets are obtained by heterologous overexpression, protein crystallizability can be improved by protein engineering [31]. An alternative to proteolysis is to identify the minimal functional fragment of the target and to design a modified gene for overexpression. As protein crystallizability is often hampered by the poor solubility of the target protein, strategies to replace hydrophobic residues (that may increase the propensity of a protein to aggregate) with hydrophilic ones can lead to diffraction-quality crystals. The solubility of the catalytic domain of HIV-1 integrase was improved by single- or multiple-point mutations of hydrophobic residues [32]: in mutants where a single hydrophobic amino acid was targeted, it was changed to lysine, and in mutants in which two or three hydrophobic amino acids were changed simultaneously, more conservative substitutions for alanine were made. From this work, a single-point mutant, F185K, showed a dramatically improved solubility and yielded X-ray-quality crystals [33]. Free surface cysteine residues may also interfere with crystallization through oxidation and the formation of intramolecular disulfide bonds. Mutation of cysteine residues to the less reactive serine can enhance crystallizability. The crystallization of human GSTO2-2 was achieved in part through a strategy whereby an initial model was created based on the homologous GSTO1-1 structure and six cysteine residues predicted to lie on the surface were mutated to serine [34]. Patel and coworkers [35] used several experimental approaches (including chemical modification and crystallography) to show that a free cysteine residue (C162) in mitogen-activated protein kinase p38α was prone to modification. As a result, a C162S mutant was prepared, which showed improved homogeneity and stability, and gave improved crystals.

In fragment screening, crystals are typically soaked in cocktails containing 3–10 compounds (see above). The compositions of the cocktails are chosen so as to contain distinctive shapes that unambiguously define them in electron density. The hit rate should be less than one compound per cocktail so as to avoid ambiguous electron density resulting from multiple hits bound with partial occupancies [17]. Some compounds may cause crystals to crack or dissolve and the risk of crystal damage increases with the number of different compounds in the cocktail. An appropriate strategy for sensitive crystals is to keep the number of fragments in each cocktail low.

Badger [36] describes further practical considerations in crystal preparation, soaking, cryoprotection, structure refinement and electron density interpretation.

### 2.3. X-ray Sources, Detectors and Robots and Software

Synchrotron radiation and robotic crystal mounting and data collection offer a significant advantage for crystallographic fragment screening [36]. Synchrotron beam-lines for macromolecular crystallography are now widespread: there are over 140 such beamlines available worldwide (http://biosync.sbkb.org). Several pharmaceutical interests operate dedicated beamlines. The Lilly Research Laboratories Collaborative Access Team (LRL-CAT) at the Advanced Photon Source, Argonne, Illinois, has been described in detail [37].

The intensity of synchrotron X-radiation facilitates short data collection times, necessitating fast-readout detectors and robotic sample-changing to increase data collection efficiency. The most popular detectors for synchrotron utilize charge-coupled device (CCD) technology. Commonly used CCDs are manufactured by Area Detector System Corporation (USA) and MarResearch (Germany). An emerging technology for diffraction data measurement is the pixel array detector (PAD). Made up of a two-dimensional array of p-n diodes joined to a readout chip [38], PADs enable readout times of less than 5 milliseconds compared with about 1 s for CCDs [37]. The ID29 beam-line at the ESRF [39] features a Pilatus 6M PAD, a 424 × 435 mm detector featuring 172 × 172 μm pixels, 2 ms readout time and is capable of recording up to 12 frames per second.

Concomitant with the deployment of robotic systems for crystal handling has been the development of control and automation software. So-called “data-collection pipelines” (DCPs) automate some or all procedures from robot sample mounting to the production of reflection data ready for structure elucidation. Robot systems for sample changing on synchrotron beam-lines include the Stanford Automated Mounter (SAM), the ACTOR™ system (Rigaku, Carlsbad, CA, USA), and the ALS Automounter. A common feature of these systems is the use of specialized cassettes for sample storage, shipping and robot handling. By way of example, SAM allows up to 288 crystals to be screened without human intervention [40]. Sample pins holding crystals are mounted in cylindrical cassettes containing 96 ports, and the robot can access three such cassettes at any one time. On synchrotron beamlines utilizing the SAM sample changer, diffraction experiments and data collection runs can be controlled at the synchrotron or remotely using the Blu-Ice/Distribute Control System [41]. At the European Synchrotron Radiation Facility (ESRF), an automatic data-collection system can track samples, control crystal mounting and alignment, determine experimental strategies based on initial images, collect the diffraction data. It can integrate, scale and reduce the experimental intensities [42].

Several programs are available for building ligands into electron density. Commercial packages include PrimeX by Schrödinger (New York, NY, USA), Rhofit by Global Phasing (Cambridge, UK) and Afitt by OpenEye (Santa Fe, NM, USA). Programs from academic laboratories include COOT [43], the Phenix suite [44] and ARP/wARP [45]. The AutoSolve platform developed by Astex Therapeutics automates X-ray data processing, structure solution and the interpretation of electron density maps [46]. For ligand fitting, AutoSolve implements a novel fitting procedure that automatically determines the compound identity (in the case of fragment cocktails) and binding mode that best explains the available electron density. Chemical information in the form of favorable interactions with the target is accounted for.

### 2.4. Potential Pitfalls in X-ray Based Screening

Historically, a crystal structure model is the result of one worker’s subjective interpretation of an electron density map [47]. Thus, the use of X-ray crystal structures in fragment screening and lead design is non-trivial and depends on the skill of the crystallographer, who should strive to model macromolecules, ligands and solvent molecules in electron density as accurately as the data will allow. While the automation of electron density maps (such as that provided by the aforementioned AutoSolve platform) is inherently objective, unambiguous interpretations of electron density maps can only be produced if recognizable shapes are present in the difference maps [46]. In section 3.3 below, we describe a recent case where cooperative binding of two fragments led to a false positive and how this was detected.

For every atom, the structure will contain the atomic coordinates and a B-factor (“temperature factor”) to model static and dynamic disorder. Note that the precision to which coordinates are reported (to 0.001 Å) is the same regardless of resolution. At resolutions lower than about 2.5 Å, the details of a protein structure (e.g., side-chain placement and the modeling of loop) may be ambiguous. The uncertainty in the position of the individual atoms could be over 0.5 Å or more below 3 Å resolution [48]. Even at moderate resolution (1.5 to 2.5 Å), uncertainties in the placement of asparagine, glutamine and histidine occur because of their internal pseudo-symmetry. In the case of asparagine and glutamine, the side-chain N and O atoms will have similar electron densities, and in the case of histidine, the N and C atoms of the imidazole ring will usually be indistinguishable (and consequently the side-chains of these residues can typically be built in two orientations). A careful investigator will account for this ambiguity by choosing a conformation on the basis of hydrogen-bond donors and acceptors surrounding the side-chains of these residues. Changes in the solvent structure that occur due to ligand binding may help to unravel unusual thermodynamic observations [49]. Interpretation of solvent binding in crystal structures becomes more difficult as resolution decreases.

Errors in the placement of ligands (including fragments) in macromolecular crystal structures can arise from several causes. Non-covalently bound ligands may exhibit greater thermal motion or conformational disorder than the surrounding protein, leading to poor electron density. Uncertainties or ambiguities in the stereochemistry or tautomeric state of a ligand may also lead to an incorrect model [50]. For fragments with internal pseudo-symmetry, considerations of hydrogen bonding, hydrophobicity, *etc*., can assist in the interpretation of ambiguous electron density. For example, the location of the nitrogen atom in a pyridine ring will be ambiguous if electron density alone is considered. (This is similar to the ambiguity noted above for the placement of histidine, asparagine and glutamine sidechains noted above.). The structures of ligands found in complexes with biological molecules may be less reliable than those of the macromolecule itself due to the fact that model building and validation tools for macromolecular structures are well developed compared to those for ligands [51]. Furthermore, geometric constraints (bond lengths, bond angles, planar and chiral restraints) applied to molecules during refinement are more thoroughly optimized for biological polymers compared with other entities. Crystal structure modeling programs, e.g., COOT [43] are able to generate geometric restraints for ligands based on SMILES strings.

Apart from pitfalls associated with the accurate interpretation of electron density, artifacts may be generated by the crystallization process itself. Potential blocking of the target site by crystal contacts can result in false-negatives upon soaking ligands. Similarly, residues surrounding the site of interest could be held in an inappropriate conformation for ligand binding, or could be blocked by other ligands. For example, crystals of the drug target, lactose dehydrogenase A were not appropriate for soaking because the active site loop was held open by crystal contacts and the substrate site was occupied by citrate from the crystallization buffer or phosphate from the purification buffer [52]. Additionally, crystals grown at extremes of pH may not yield ligand-binding modes observed at physiological pH due to protonation/deprotonation of susceptible side-chains. For example, at pH 3, the protonation of aspartate and glutamate side-chains will be significant and will alter the charge and hydrogen-bonding characteristics of the protein. Some structures undergo pH dependant conformational changes. For example, the severe acute respiratory syndrome coronavirus (SARS-CoV) main protease shows substantial differences at pH 6.0, 7.6 and 8.0 [53].

## 3. Examples of Crystallography in FBDD

In this section, we describe some recent examples of FBDD against protein targets and the techniques (in addition to crystallography) brought to bear to screen fragments and assay binding. Table 1 lists several recent projects and the various techniques used for screening and assays. The use of FBS in lead development of inhibitors of β-secretase (Section 3.1) represents a classic example of crystallography in FBDD and will be discussed in detail. In Section 3.2, fragment screening against phosphodiesterase 4—where crystals were not of sufficient quality for direct use in screening—is described. Here, a new technology for high-throughput calorimetry (enthaply arrays) was used for pre-screening fragments for crystallography. Fragment screening against human phenylethanolamine *N*-methyltransferase by crystallography (Section 3.3) provides an interesting case of cooperative fragment binding (from fragments soaked into crystals in cocktails). Finally, the complementary use of NMR and crystallography in the development of Hsp90 inhibitors is described (Section 3.4).

### 3.1. β-Secretase

**Figure 1:**
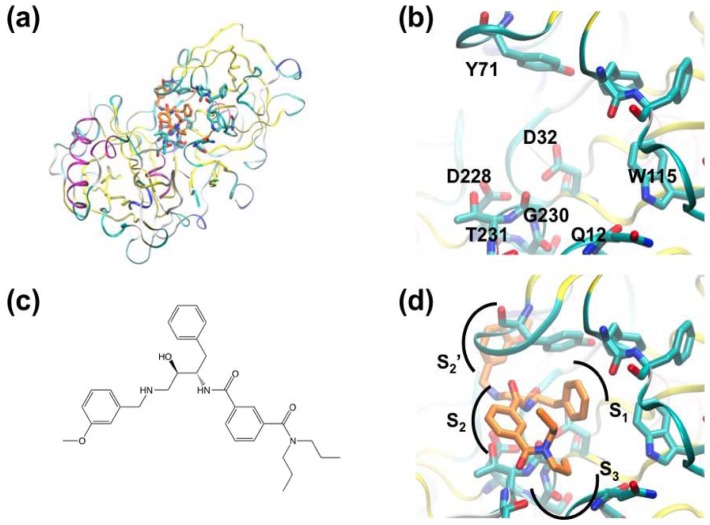
Structure Beta secretase-1 (BACE-1). (**a**) Overall fold showing location of active site; (**b**) Active site residues; (**c**) Hydroxyethylamine-based peptidomimetic inhibitor; (**d**) Same compound shown in BACE-1. Sub-sites are labeled according to the amino-acids either side of the cleavage site (S_2_, S_1_, S_1_′, S_2_′, *etc*.).

Amyloid plaques and associated neurofibrillar tangles are known to occur in the brains of Alzheimer’s disease (AD) patients. These plaques are composed of β-amyloid peptides derived from amyloid precursor protein by the activity of β- and γ-secretases. β*-*Secretase is known to cleave amyloid precursor protein to yield the *N*-terminus of the β*-*amyloid peptides. Beta secretase-1 (BACE-1) is an aspartyl protease responsible for β*-*amyloid production and this enzyme is a potential therapeutic target for treatment of AD. Figure 1 shows BACE and its active site in complex with a peptidomimetic inhibitor [67].

**Figure 2:**
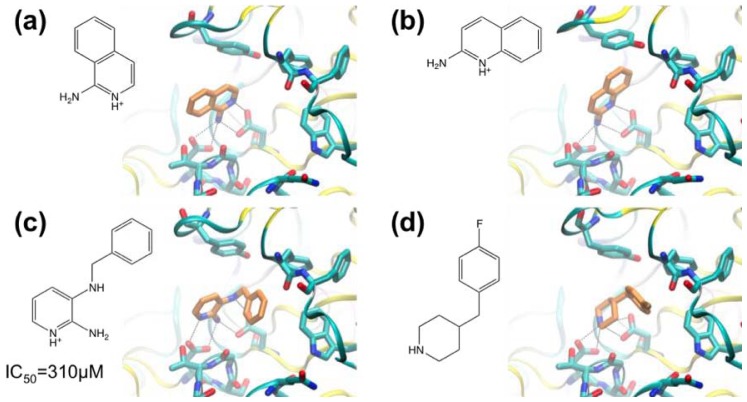
Crystal structures of fragment complexes with BACE-1. The chemical structures of the protonated forms of the compounds are shown, and, where determined, IC_50_ values are shown. (**a**) 1-aminoisoquinoline; (**b**) 2-aminoquinoline; (**c**) 2-amino-3- (benzylamino)pyridine; (**d**) 4-(4-fluorobenzyl)piperidine.

Murray and co-workers [54] have used crystallography for FBS against BACE-1. A library containing 347 fragments was screened in cocktails containing six compounds. Two hits containing aminopyridine motifs were found that formed hydrogen bonds with catalytic aspartate residues D32 and D228 (Figure 2a,b). The hits form nearly identical interactions with BACE-1. In both cases the amine groups form hydrogen bonds with D228 and the protonated pyridine group donates a hydrogen bond to D32. This charged bidentate interaction had not been described previously in aspartyl proteases. The hydrophobic S_1_ and S_3_ pockets adjacent to these ligands are essential for substrate peptide binding. Murray and co-workers sought to identify further fragments based on the aminopyridine motif seen in Figure 2a,b that would allow access to the S_1_ and S_3_ regions. Docking calculations were used to select another 65 compounds for screening, including a focused set of fragments containing 2-aminopyridine motifs, and cyclic secondary amines (shown to interact with catalytic aspartate residues in renin) were also selected. Compounds were docked into multiple protein conformations of BACE-1 using a modified version of the GOLD software [68]. Thus, fragments were ranked and 65 were selected for crystallographic screening against BACE-1. 2-Amino-3-(benzylamino)pyridine (Figure 2c) was found to bind with an IC_50_ of 310 μM (estimated from a BACE assay utilizing a fluorescently labeled peptide). Interestingly, the aminopyridine moiety of the compound changed orientation relative to the fragment hits (*cf*. Figure 2a,b), allowing an additional hydrogen bond between the 3-amino group and D32. A cyclic secondary amine, 4-(4-fluorobenzyl)piperidine (Figure 2d) was also identified from virtual screening.

**Figure 3:**
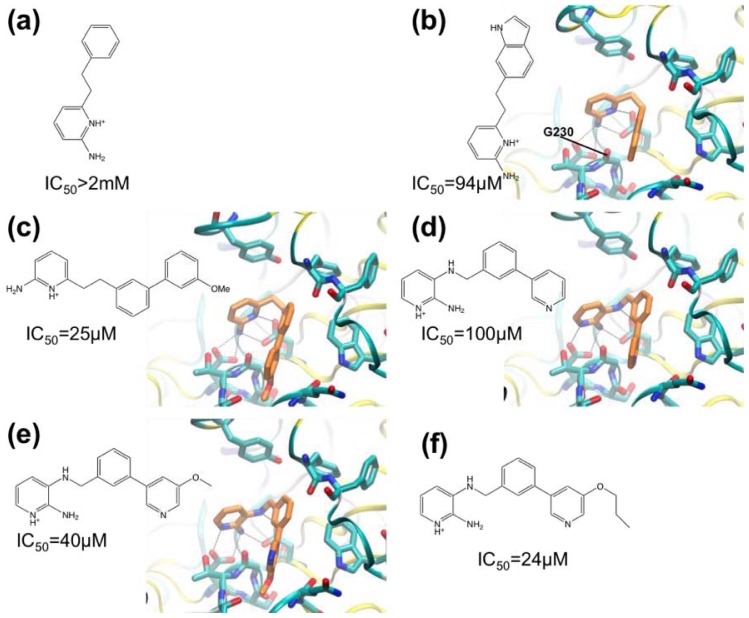
Chemical structures of BACE-1 inhibitors developed from fragment hits. The structural formulae of the protonated forms of the compounds bound are shown, and, where determined, IC_50_ values and crystal structures of complexes with BACE-1 are shown. (**a**) 2-amino-6-phenethylpyridine; (**b**) 2-(2-(1*H*-indol-6-yl)ethyl)-6-aminopyridine; (**c**) 2-amino-6-(2-(3′-methoxy-[1,1′-biphenyl]-3-yl)ethyl)pyridine; (**d**) 2-amino-3-((3- (pyridin-3-yl)benzyl)amino)pyridine; (**e**) 2-amino-3-((3-(5-methoxypyridin-3-yl)benzyl) amino)pyridine; (**f**) 2-amino-3-((3-(5-propyloxypyridin-3-yl)benzyl)amino)pyridine.

In the companion paper, Congreve and coworkers [55] describe the synthesis of series of 3- and 6-substituted 2-aminopyridine derivatives with a view to making compounds that occupy the S_1_ pocket in addition to blocking the catalytic aspartate residues. A phenyl group was predicted to make suitable hydrophobic interactions with the S_1_ region and the compound phenylethyl-2-aminopyridine (Figure 3a) was synthesized (IC_50_ > 2 mM). The next compound synthesized was the indole-substituted aminopyridine (IC_50_ = 94 μM) (Figure 3b), which displayed more favorable interactions with the S_1_ pocket compared with the phenyl-substituted compound. A polar interaction was observed between the indole NH and the carbonyl oxygen of residue G230. The greatly improved IC_50_ value indicated that the S_1_ was a useful area to target in the next iteration of design. Derivatives were synthesized including one in which a 3-methoxy-biaryl group replaced the indole substituent (IC_50_ = 25 μM) (Figure 3c) which also made favorable interactions with the pocket; the biaryl motif was judged suitable for further modification. By combining the biaryl moieties of these hits with the 2-amino-3-(benzylamino)pyridine hit (Figure 2c), biaryl substituted 2,3-diaminopyridines were synthesized to better fit the S_1_-S_3_ pocket (Figure 3d–f). The 3-pyridyl group introduced into these compounds appeared to form favorable contacts with the pocket. The best compound in this series had an *n*-propyloxy group in place of the methoxy group (Figure 3f) (IC_50_ = 24 μM). This compound was considered to be a high quality lead and was prioritized for further development.

**Figure 4:**
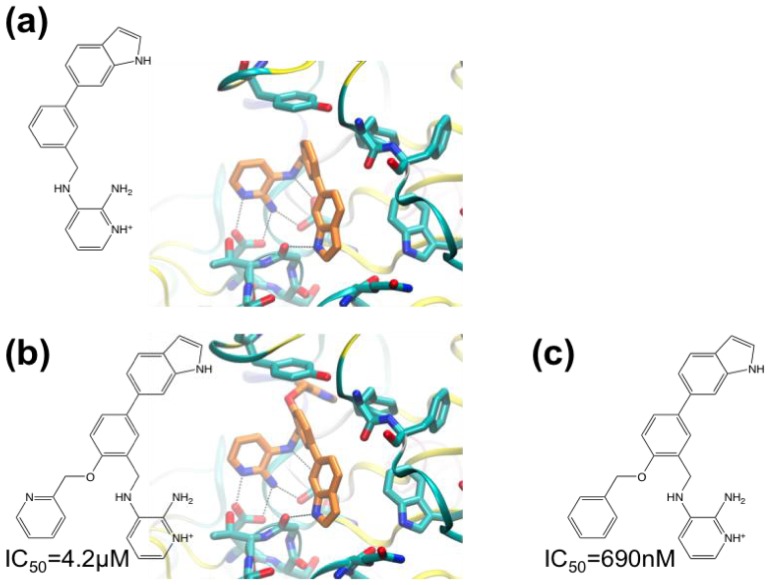
Chemical structures of high-affinity BACE-1 inhibitors. The structural formulae of the protonated forms of the compounds bound are shown, and, where determined, IC_50_ values and crystal structures of complexes with BACE-1 are shown. (**a**) 3-((3-(1*H*-indol-6- yl)benzyl)amino)-2-aminopyridine; (**b**) 3-((5-(1*H*-indol-6-yl)-2-(pyridin-2-ylmethoxy) benzyl)amino)-2-aminopyridine; (**c**) 2-amino-3-((2-(benzyloxy)-5-(1*H*-indol-6-yl)benzyl) amino)pyridine.

Compounds with indolyl groups substituted for the 3-pyridyl ring were made to better occupy the S_3_ pocket (Figure 4a–c). The indolyl group in these compounds occupied the S_3_ pocket and formed a new hydrogen bond between the indolyl-NH and the G230 carbonyl group (IC_50_ = 9.1 μM). Next, the ortho-position of the phenyl ring (adjacent to the methylene linker), was targeted with a view to occupying the S2′ sub-site. Two ligands were made containing a 2-pyridinylmethyloxy group or benzyloxy group (Figure 4b,c). The benzyloxy-containing compound was too insoluble to allow high-quality crystal structure determination, but it is the most potent inhibitor with IC_50_ of 690 nM.

These structures show the evolution of fragment hit to nanomolar-inhibitor. These compounds include a binding motif not previously observed in aspartic protease inhibitors: the aminopyridine motif that interacts with the catalytic aspartate residues.

### 3.2. Phosphodiesterase 4A

**Figure 5:**
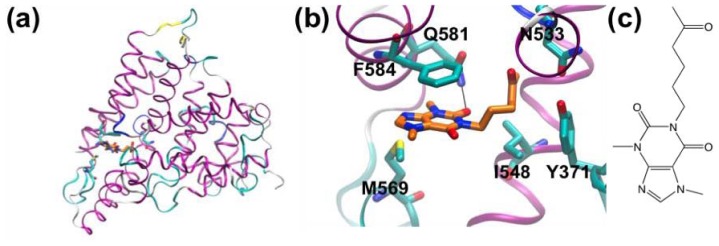
(**a**) Structure of human PDE4A in cartoon form; (**b**) Active-site of PDE4A in complex with inhibitor pentoxifylline; (**c**) Structure of pentoxifylline.

The cAMP-degrading phosphodiesterase 4 (PDE4) family of enzymes is a potential target for therapeutics for the treatment of chronic obstructive pulmonary disease (COPD), asthma, depression and neurodegenerative diseases. Human PDE4A (Figure 5) is difficult to crystallize, making screening by crystallography impractical. Recht and co-workers [57] have used enthalpy arrays (*i.e.*, arrays of nanocalorimeters) to perform enzyme activity-based fragment screen for inhibitors of PDE4A activity.

**Figure 6:**
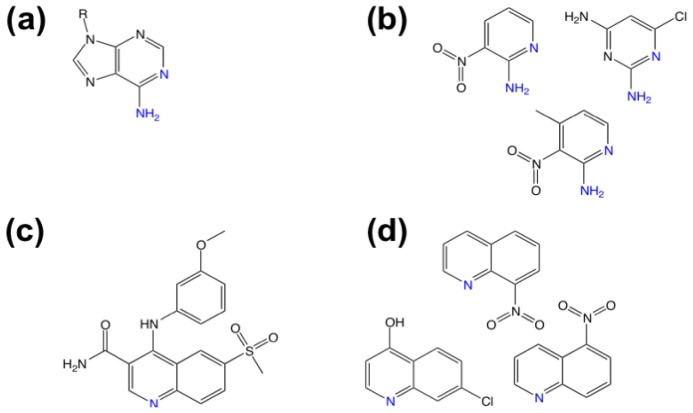
(**a**) Adenine component of cAMP; (**b**) Purine-like hits; (**c**) 4-[3-(methoyxphenyl)amino]-6-(methylsulfonyl)quinoline-3-carboxamide; (**d**) quinoline-like hits. Analogous nitrogen atoms are highlighted in blue.

The fabrication of 96-channel enthalpy arrays for the measurement of thermodynamic and kinetic parameters of molecular interactions was described by Torres and co-workers [69]. The array uses small sample volumes (250 nL) and short assay times (typically 5 to 10 min) not possible in conventional calorimetry. The assay used for PDE4 was based on the hydrolysis of cAMP measured at 21 °C. The assay was validated using the nonselective phosphodiesterase inhibitors pentoxifylline (Figure 5c) and 3-isobutyl-1-methylxanthine (IBMX). Initially, a 160-compound fragment library was screened for competitive inhibitors. From the calorimetric data, the change in *K_M_* in the presence of the fragment was used to determine *K_I_*. Eleven fragments with *K_I_* values between 320 and 1800 μM were selected for crystallography, and complexes with fragments were obtained by co-crystallization with diffraction quality apparently dependent on ligand potency. High-quality diffraction was obtained with one potent ligand, pentoxifylline (*K_I_* 72 μM) (Figure 5b,c). It is noteworthy that six of the fragment hits contain functional groups that contain purine-like or quinoline-like features (Figure 6).

While the natural substrate (cAMP) contains a purine ring (Figure 6a), the known PDE inhibitors including 4-[3-(methoxyphenyl)amino]-6-(methylsulfonyl)quinoline-3-carboxamide contain a quinoline system (Figure 6c). Although it was not possible to obtain crystal structures of PDE4A with all fragments, complexes of PDEs with substrates and inhibitors suggest a common binding mechanism whereby ligand nitrogen atoms accept a hydrogen bond from active-site residue Q581.

The use of enthalpy arrays in fragment screening has several benefits: the number of X-ray structures to be determined is reduced (which may be necessitated where structural characterization is challenging), and the type of binding (competitive, uncompetitive, *etc*.) can be elucidated.

### 3.3. A Case of Cooperative Fragment Binding: hPNMT

**Figure 7:**
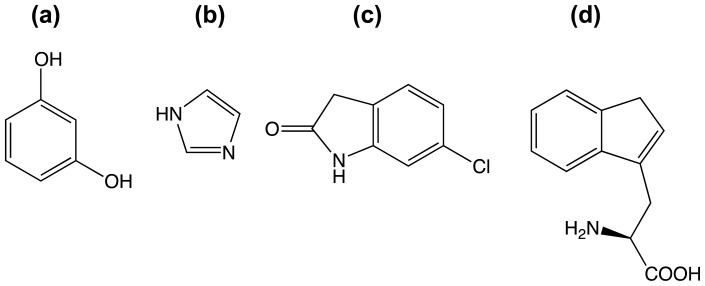
(**a**) Resorcinol; (**b**) Imidazole; (**c**) 6-Chlorooxindole; (**d**) (*S*)-2-amino-3-(1*H*inden- 3-yl)propanoic acid.

Human phenylethanolamine *N*-methyltransferase (hPNMT) catalyses the last step in adrenaline synthesis: the conversion of noradrenaline to adrenaline, utilizing *S*-adenosyl-methionine (SAM) as the methyl-group donor. Adrenaline in the central nervous system is implicated in a range of physiological and pathological conditions including Parkinson’s and Alzheimer’s diseases and PNMT inhibitors are of potential therapeutic value. Drinkwater *et al.* [70] used 96 cocktails of four fragments each (a total of 384 compounds) for screening against hPNMT by crystallography. A cocktail was considered to be a false positive if examination of the electron density failed to identify a specific fragment, and if no density was observed when fragments were soaked into the crystal separately. A total of 12 compounds were ultimately identified, binding in the noradrenaline-binding site. ITC was used to confirm the hits, and 3 compounds were ruled out. Subsequent analysis revealed that some of the false-positive hits from cocktail soaking was due to cooperative binding: Nair and co-workers used molecular dynamics (MD) to show that one false positive was actually two fragments cooperatively bound in the active-site [56]. Figure 7 shows the compounds in one of the cocktails used to soak hPNMT crystals.

**Figure 8:**
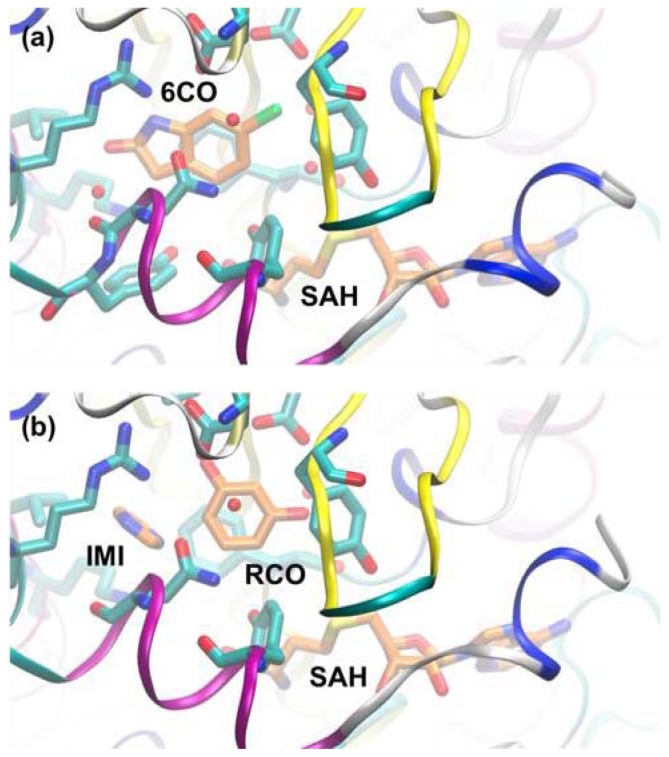
(**a**) PNMT with 6-chlorooxindole (6CO) and *S*-adenosyl-homocysteine (SAH) modelled in the active site (based on PDB entry 3KPY); (**b**) Same structure after reassignment of density to imidazole (IMI) and resorcinol (RCO) (based on PDB entry 4DM3). Protein backbone is shown in ribbon form, with residues shown in stick form. Ligands are drawn in stick form with carbon atoms colored orange.

Electron density was initially interpreted as compound 6-chlorooxindole (Figure 7c), but binding could not be validated by ITC [70]. Nair *et al.* performed MD simulations of the PNMT/ 6-chlorooxindole complex, showing that the compound was ejected from the binding pocket on 50−100 ps of simulation [56]. A series of simulations and re-assessment of the electron density led to a model in which compounds shown in Figure 7a,b are bound cooperatively. The structures corresponding to these structural interpretations are shown in Figure 8.

### 3.4. Heat Shock Protein 90 (Hsp90)

Hsp90 is an ATP dependent molecular chaperone that modulates protein stability and is a key component of the heat-shock response [71]. Many client proteins of Hsp90 have been identified and several are involved in cellular signaling (e.g., kinases and transcription factors) and play critical roles in cancer progression [72]. Over-expression of Hsp90 has been demonstrated in cancer types such as advanced malignant melanoma, oesophageal squamous cell carcinoma, non-small cell lung cancer and pancreatic carcinoma [73]. Hsp90 is an attractive target for chemotherapy: inhibition of its activity affects multiple signaling pathways or cellular processes required for cancer cells to survive under stress [74]. Furthermore, Hsp90 inhibitors work synergistically with several other drugs in the treatment of both solid tumors and leukemias. Inhibiting Hsp90 with small molecules has been a popular area of research interest over the last decade and progress had been made in diversifying available chemotypes [75].

**Figure 9:**
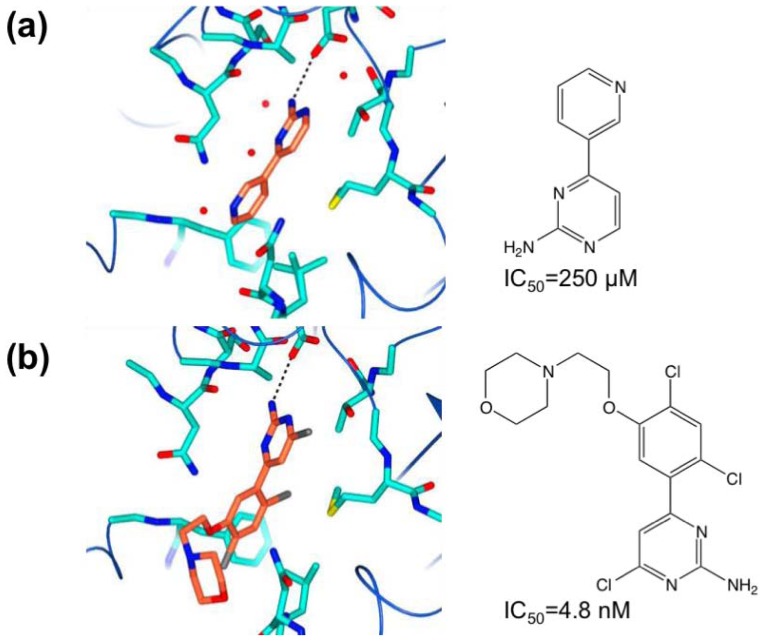
(**a**) Hsp90 with pyrimidine fragment bound, and (**b**) with 4-chloro-6-(2,4- dichloro-5-(2-morpholinoethoxy)phenyl)pyrimidine-2-amine bound.

Murray and co-workers have applied fragment-based screening to Hsp90 using NMR, ITC and X-ray crystallography [58,59]. About 1600 compounds were screened against Hsp90 in cocktails using ligand observed NMR via waterLOGSY. Favorable compounds were further characterized by an NMR assay measuring displacement by fragments of ADP in Hsp90’s nucleotide binding site. Based on the NMR assay and considerations of structural diversity, 125 fragments were selected for crystallography, resulting in 26 crystal structures. One of these was an aminopyrimidine compound (Figure 9a) with measured affinity of 250 μM (estimated by ITC). The pyrimidine moiety binds in a structurally analogous way to the purine ring of ADP. The molecule forms an extensive network of hydrogen bonds with the side chain of D93 and water molecules. Development of the fragment proceeded by expansion of the compound to better fill the lipophilic pocket occupied by the pyridine moiety and by reduction of the internal strain due to the torsion angle of the two rings. The crystal structure (Figure 9a) shows that the pyridine and pyrimidine rings are rotated by 47.6° along their connecting bond, whereas the optimal geometry for such systems should be close to 0°. Structure-activity relation (SAR) analysis with quantum mechanical calculation of the torsional contribution and subsequent modification led to compounds with the pyridine ring substituted for phenyl groups with methoxy- and chloro- substitutions at the 2- and 6-positions. These substitutions both relieved the strain and better filled the proximal lipophilic pocket. A 4-chloro-substitution on the ring further improved occupancy of the lipophilic pocket. The solubility of the compounds was increased in order to improve cell-based activity. The result of these efforts was the incorporation of an *N*-ethylmorpholino-group at the 5-position of the phenyl ring. The resulting compound, shown in Figure 9b, has an IC_50_ of 4.8 nM (ITC estimate). It is noteworthy that the orientation of this compound is essentially the same as the starting fragment while the interactions are maintained.

**Figure 10:**
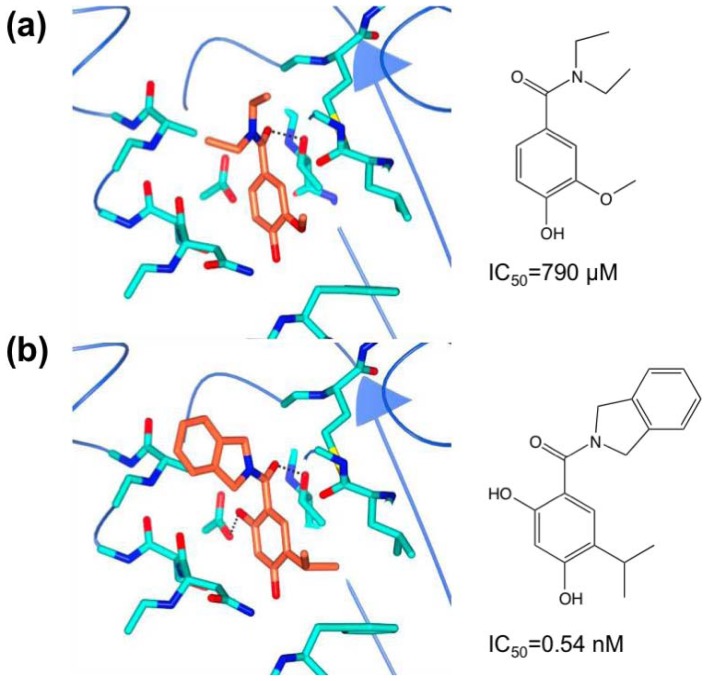
(**a**) Hsp90 with phenol-based fragment bound, and (**b**) with (2,4-dihydroxy-5- isopropylphenyl)(isoindolin-2-yl)methanone bound.

Similar strategies were applied to a phenol-based fragment hit (Figure 10a, IC_50_ = 790 μM by ITC). The structure of the complex of this fragment with Hsp90 indicated that the methoxy-group could be replaced with substituents that occupied the proximal lipophilic pocket. A *tert*-butyl or isopropyl group at this position improved the affinity 100-fold. Scaffolds with these substitutions were advanced to synthetic efforts focused on the diethylamide group, while the carbonyl group was kept to maintain the hydrogen-bond with an adjacent threonine residue and adjacent water molecules. An isoindoline group replacing the diethylamide group delivered an affinity improvement of several-hundred-fold. Eventually the phenol core was converted to a resorcinol core due to its similarity with the natural product radicicol, a known Hsp90 inhibitor. The final lead (Figure 10b, IC_50_ = 0.54 nM by ITC) was further developed into drug candidates under clinical trial [59].

## 4. Conclusions

FBDD is a flexible and potent tool for the creation of small molecules with potential for development into drugs. Coupled with high-resolution structures of protein–fragment complexes, focused strategies for medicinal chemistry for fragment evolution can be derived. There are many possible work-flow designs for fragment screening that can be utilized in drug discovery and contemporary efforts typically use more than one biophysical method. Thanks to advances in automation software and robotics, crystallography can be used as the primary screening technique and for the determination of the topology of the binding of fragment to the target protein. However, to determine binding affinities of hits, other techniques should be applied. The general ease of access to synchrotron radiation and attendant robotic infrastructure makes this approach broadly accessible. Here we have described four cases where crystallography has contributed to FBS and where successes and pitfalls are highlighted. In the case of BACE-1 inhibitors, crystallography was straightforward and contributed to the development of nanomolar IC_50_. In the case of PDE4A, crystallography was challenging and the screening program benefited from the new enthalpy array technology to pre-select fragments for further study. Finally, FBS against Hsp90 revealed multiple hits that gave rise to alternative nanomolar-IC_50_ inhibitors. FBS against hPNMT demonstrated a case of cooperative fragment binding and its diagnosis by MD simulations. In spite of some disadvantages described in the article, crystallography plays a central role in structural biology and drug discovery processes. The ultimate value of X-ray crystallography is in the visualization of fragment binding details that can indicate vectors for the expansion and evolution of hits. Orthogonal screening techniques are of value for ruling out false positives.

## Figures and Tables

**Table 1:** Recent examples of fragment-based screening.

Reference	Article title	Target protein	Primary/secondary FBDD screening method	Binding/activity assay
[54,55]	Application of Fragment Screening by X-ray Crystallography to the Discovery of Aminopyridines as Inhibitors of -Secretase	β-Secretase	X-ray crystallography	Fluorescence-based activity assay
[56]	Missing fragments: detecting cooperative binding in fragment-based drug design	hPNMT	X-ray crystallography	ITC/Molecular dynamics free energy calculation
[57]	Fragment-based screening for inhibitors of PDE4A using enthalpy arrays and X-ray crystallography	Phosphodiesterase 4A	High-throughput calorimetry/X-ray crystallography	High-throughput calorimetry
[58,59]	Fragment-Based Drug Discovery Applied to Hsp90. Discovery of Two Lead Series with High Ligand Efficiency	Hsp90	NMR/X-ray crystallography	ITC/Bioassay
[60]	Fragment-Based Discovery of Bromodomain Inhibitors Part 1: Inhibitor binding modes and implications for lead discovery	Bromodomain	Fluorescence anisotropy assay/X-ray crystallography	Fluorescence anisotropy assay
[61]	Fragment-Based Discovery of Bromodomain Inhibitors Part 2: Optimization of Phenylisoxazole Sulfonamide	Bromodomain/AcK pocket	Fluorescence anisotropy assay/Modelling X-ray crystallography	SPR/Thermal shift assay
[62]	Structure-based design of potent and ligand-efficient inhibitors of CTX-M class A β-lactamase	β-lactamase CTX-M	Docking/X-ray crystallography	UV-absorbance based bioassays/Antibacterial activity
[63]	Discovery of 1,2,4-triaine derivatives as adenosine A2A antagonists using structure based drug design	Adenosine A2 receptor	Docking/X-ray crystallography	SPR
[64]	Discovery and Optimization of New Benzimidazole- and Benzoxazole-Pyrimidone Selective PI3Kβ Inhibitors for the Treatment of Phosphatase and TENsin homologue (PTEN)-Deficient Cancers	PI3K	*In vitro* enzyme assay/Cell based assay X-ray crystallography	*In vitro* enzyme assay/Cell-based assay
[65]	Synthesis, Structure–Activity Relationship Studies, and X-ray Crystallographic Analysis of Arylsulfonamides as Potent Carbonic Anhydrase Inhibitor	Carbonic anhydrases	Docking/X-ray crystallography	Stopped-flow kinetic assay
[66]	Implications of Promiscuous Pim-1 Kinase Fragment Inhibitor Hydrophobic Interactions for Fragment-Based Drug Design	Pim-1 Kinase	Docking/X-ray crystallography	Mobility shift assay
